# Pediatric Multisystem Inflammatory Syndrome in Children as a Challenging Problem for Pediatric Surgeons in the COVID 19 Pandemic—A Case Report

**DOI:** 10.3389/fped.2021.677822

**Published:** 2021-06-11

**Authors:** Beata Jurkiewicz, Magdalena Szymanek-Szwed, Piotr Hartmann, Joanna Samotyjek, Eliza Brędowska, Joanna Kaczorowska, Ewa Wajszczuk, Martyna Twardowska-Merecka, Joanna Cybulska

**Affiliations:** ^1^Department of Pediatric Surgery and Pediatric Urology, Medical Center of Postgraduate Education, Dziekanów Leśny, Poland; ^2^Department of Pediatrics, Children's Hospital, Dziekanów Leśny, Poland

**Keywords:** pediatric inflammatory multisystem syndrome, appendectomy, coronavirus disease-19, children, case report

## Abstract

The first cases of severe acute respiratory syndrome coronavirus-2 (SARS-CoV-2) infection were identified at the end of 2019 and, in the next few months, coronavirus disease (COVID-19) spread throughout the world. Initially, it was believed that this disease mainly affected elderly individuals with comorbidities, in whom respiratory failure often occurs. It was believed that children fell ill from the infection more often, although the course of infection in the vast majority of pediatric cases has been asymptomatic or mildly symptomatic. In April and May 2020, the first report of a rapidly progressing disease, similar to Kawasaki syndrome, was found in children who had been infected with SARS-CoV-2. Shortly thereafter, children with symptoms of pediatric inflammatory multisystem syndrome (PIMS-ST [temporally associated with SARS-CoV-2 infection]) began presenting to pediatric hospitals around the world. The syndrome has a mortality rate of up to 2%. Symptoms of PIMS-TS include those that may suggest the need for surgical treatment (severe abdominal pain with the presence of peritoneal symptoms, ascites, high levels of inflammatory markers, intestinal inflammation, and appendages revealed on ultrasound examination). However, there are few reports addressing surgical cases associated with this condition. The authors present a case involving an 11-year-old boy who was admitted to hospital with severe abdominal pain and underwent surgery for symptoms of peritonitis and was diagnosed with PIMS in the post-operative period. Due to the large number of illnesses caused by SARS-CoV-2 infection in recent months, the diagnosis of PIMS-TS/MISC should be considered in the differential diagnosis of acute abdominal symptoms, especially in atypical courses and interviews indicating exposure to SARS-CoV-2.

## Introduction

The first cases of coronavirus disease (COVID-19), caused by infection with severe acute respiratory syndrome coronavirus-2 (SARS-CoV-2), were reported at the end of 2019 in the city of Wuhan, Hubei Province, China. Within a few months, COVID-19 spread around the world (1). Initially, it was believed that COVID-19 mainly affects the elderly and those with specific comorbidities (2, 3).

According to data from the American Pediatric Society, childhood cases constitute ~12% of the total. Initially, it was reported that, apart from the fact that children are less affected, the course of infection in the pediatric population is asymptomatic or mildly symptomatic in the vast majority of cases. Hospitalization rates for COVID-19 in pediatric patients were <5% of those for adults (4).

In the pediatric population, the course of the disease is often asymptomatic, and if respiratory symptoms do emerge, they are usually not severe (5). However, in April and May 2020, the first reports from the United States and the United Kingdom regarding the course of COVID-19 in children appeared and initially suggested an atypical course resembling Kawasaki disease or toxic shock syndrome (6). Based on previous observations and the experience of many centers, the concept of pediatric multisystem inflammatory syndrome temporarily associated with SARS-CoV-2 infection was presented, [PIMS-TS—pediatric multisystem inflammatory syndrome in children—UK Royal College of Pediatrics and Child Health (RCPCH)/MIS-C—multisystem criteria] inflammatory syndrome in children— (criteria from the World Health Organization and Centers for Disease Control and Prevention) (Table 1).

**Table 1:** MIS-C criteria from the World Health Organization.

**Patient aged 0–19 years with fever ≥ 3 days**
+2 of the following symptoms • Acute gastrointestinal disorders (diarrhea, vomiting, abdominal pain) • Rash or bilateral non-purulent conjunctivitis or muco-cutaneous inflammation signs (oral, hands or feet) • Hypotension or shock • Features of myocardial dysfunction, pericarditis, valvulitis, or coronary abnormalities • Evidence of coagulopathy
+ Elevated markers of inflammation such as ESR, C-reactive protein, or procalcitonin
+ No other obvious microbial cause of inflammation, including bacterial sepsis
+ Evidence of COVID-19 **(**RT-PCR, antigen test or serology positive), or likely contact with patients with COVID-19

Symptoms of PIMS-TS/MIS-C are presented in Table 1.

Laboratory investigations have revealed high serum levels of C-reactive protein (CRP) and ferritin, as well as lymphopenia, neutrophilia, hypercoagulability, hyponatremia, and hypoalbuminemia. The most serious complications include left ventricular muscle insufficiency, with a decrease in ejection fraction, the appearance of coronary aneurysms, and embolic complications (7, 8). The pathogenesis of the syndrome is not fully understood. Only ~45% of patients exhibit positive polymerase chain reaction (PCR) results for SARS-CoV-2, although the presence of antibodies is found in 75%. Symptoms of the syndrome often appear only several weeks after contact with a sick person or an infection transmitted from an asymptomatic or slightly symptomatic individual. Pre-disposing factors include age (~≥9 years), male sex, obesity, and African–American ethnicity (7, 8). The syndrome has a mortality rate of up to 2%. Symptoms of PIMS-TS include those that may suggest the need for surgical treatment (severe abdominal pain with the presence of peritoneal symptoms, ascites, high levels of inflammatory markers, intestinal inflammation, and appendages revealed on ultrasound examination). However, there are few reports addressing surgical cases associated with this condition.

We present a case involving a boy treated in the Department of Pediatric Surgery and Pediatric Urology at the Medical Center of Post-graduate Education. It is important to emphasize that, in this patient, PIMS-TS was diagnosed only during the post-operative course. Therefore, physicians should be aware of the possibility of an atypical course of complications of SARS-CoV-2 infection in patients hospitalized for symptoms of “acute abdomen.”

## Case Study

**Figure 1:**
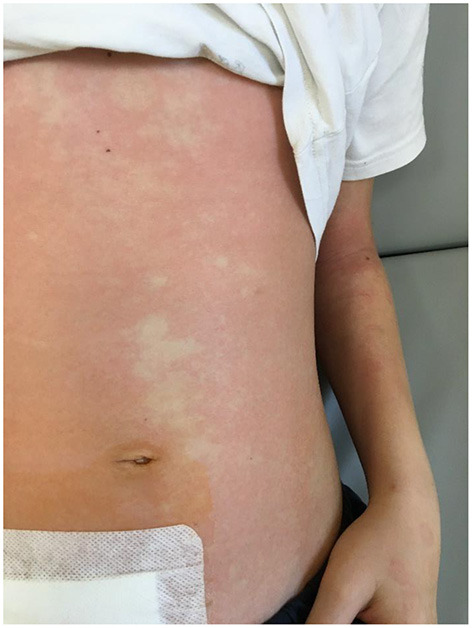
Skin lesions.

A boy aged 11 years and 3 months was admitted to the hospital due to a 4-day history of worsening abdominal pain and low-grade fever (37.6°C). The pain was initially localized to the epigastric region, and then increased in the right iliac fossa during movement. Decreased appetite was also observed. During outpatient treatment, he received paracetamol, drotaverine, and a probiotic, which did not result in improvement. On the day of admission, outpatient laboratory investigations revealed an elevated CRP level [7.58 mg/dL (75.8 mg/L)] and a normal leukocyte count, with no other significant abnormalities, including the urinalysis results. After pediatric consultation, the boy was admitted to the hospital with suspected appendicitis. An interview revealed that the child was generally healthy. Before admission to the hospital, he had not taken any medications on a regular basis and had not been previously hospitalized. He was vaccinated according to PSO, Rotarix, Prevenar 13. On admission, the patient was in a good general condition. Physical examination revealed compressive soreness in the right iliac fossa, with clear muscular defense and positive peritoneal symptoms, without any other abnormalities. Laboratory investigations performed on admission revealed elevated CRP level (66.8 mg/L), borderline leukocyte count (4.04 × 10^3^/μL), neutrophilic smear (70.7%), lymphopenia (0.77 × 10^3^/μL), and normal red cell system and platelet count. General urine examination revealed ketonuria, slight proteinuria (40 mg/dL), increased urobilinogen, borderline leukocyturia (3–5 hpf), and erythrocyturia (4–6 hpf). Ultrasound examination revealed the presence of vesicular fluid and thickening of the cecum wall. Real-time PCR testing of a nasopharyngeal swab was negative for SARS-CoV-2. Due to the characteristic history and physical examination, acute appendicitis was suspected and the patient was referred for emergency laparotomy. Intraoperatively, an unchanged appendix and a significant amount of serous fluid were found. Classic appendectomy was performed, the peritoneal cavity was drained, and no other source of peritoneal inflammation was found. The intestines were normal, and the mesenteric lymph nodes were not enlarged. The patient's early post-operative course was uneventful. Perioperatively, the patient received antibiotic prophylaxis with cefazolin (5 doses in total) due to large amount of fluid in abdominal cavity. Good tolerance of an easily digestible diet was observed. After the surgery, the boy experienced watery stools (a total of up to 4 days) without pathological impurities. Upon suspecting bacterial infection of the gastrointestinal tract, antibiotic therapy was modified on day 2 of hospitalization, and cefazolin was discontinued and intravenous cefotaxime was empirically administered. Before treatment modification, serology for yersiniosis was performed, and feces were sent for culture. The boy complained of persistent pain and low-grade fever. In the following days, the results of stool culture and serological tests for yersiniosis were negative. On day 4 of hospitalization, a single ring-shaped skin lesion was observed. Suspected allergic background was treated with a second-generation antihistamine (levocetirizine). The next day, a fever of >39°C was observed. The boy's condition deteriorated, and he reported feeling unwell and weak. Control laboratory investigations revealed an increase in inflammatory marker levels (CRP, 137.6 mg/dL; procalcitonin, 1.96 ng/mL), with normal peripheral blood counts and leukocytes count, neutrophil smear of 86.2%, signs of normocytic anemia (hemoglobin, 10.4 g/dL; mean corpuscular volume, 79.5 fL), and normal platelet count. Antibiotic therapy was extended to include metronidazole and intravenous amikacin. A control ultrasound examination of the abdominal cavity 5 days after appendectomy revealed an increased amount of fluid with increased echogenicity in the bladder area, thickened walls of the cecum and terminal intestine, and enlarged mesenteric nodes (up to 20 × 14 mm) in the area of the removed appendix. Owing to the ultrasound image suggesting the possibility of purulent lesions, computed tomography (CT) examination of the abdominal cavity was performed and revealed traces of fluid in the pleural cavities, the presence of fluid in the peritoneal cavity, interloop and bladder fluid (~150 mL), thickened walls of the cecum (up to 19 mm), thickened wall of the end intestine (up to 8 mm), without the presence of free gas in the peritoneal cavity, and numerous lymph nodes at the cecum and on the iliopsoas muscle (up to 28 mm). Due to the large amount of fluid and high fever, suggesting the formation of purulent lesions in the abdominal cavity, a decision was made to repeat the laparotomy. During the procedure, a large amount of slightly cloudy serous fluid was aspirated from the peritoneal cavity, which was sent for culture (negative), and a drain was inserted into the bladder area. Intraoperative images of the intestines did not concur with the ultrasound and CT descriptions; no inflammatory changes in the intestinal wall were confirmed. Control blood tests revealed hyponatremia (134 mmol/L), hypokalemia (3.2 mmol/L), and hypoalbuminemia (3.1 g/dL), with a decreased total protein concentration (5 g/dL). To determine the cause of the boy's poor general condition, diagnostics were directed toward a proliferative etiology. Normal tumor marker (carcinoembryonic antigen and alpha-fetoprotein) levels were also determined. On day 1 after the repeat laparotomy, fever persisted. Additionally, a reddened appearance was observed on the skin of the trunk. The annular lesions intensified and fused to form a “garland” (Figure 1).

**Figure 2:**
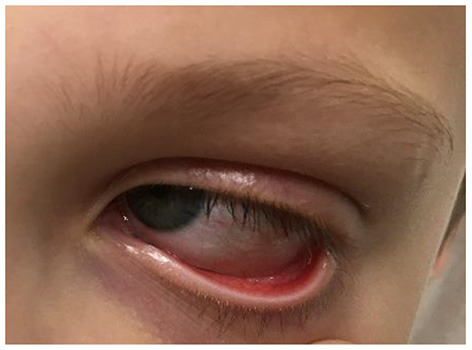
Conjunctivitis.

Additionally, the patient developed bilateral non-pyrogenic conjunctivitis, as well as chapped and reddened lips (Figure 2).

He exhibited a temperature of 40°C despite a constant supply of paracetamol. During consultation with pediatrics, information regarding exposure to SARS-CoV-2/COVID-19 ~1 month before the onset of disease symptoms was obtained. This explained the high positivity for SARS-CoV-2 antibodies [cut-off index (COI), 102 (normal, <0.99)]. In control laboratory tests, a further increase in inflammatory markers was observed [CRP, 176.1 mg/L; procalcitonin, 2.97 ng/mL; ferritin, 301 ng/mL (normal, <124 ng/mL)]. Moreover, increased hypokalemia, hypoalbuminia, and features of slight disorders of the coagulation system [international normalized ratio, 1.34 (normal, 0.85–1.25); D-dimer, 4,460 μg/dL (normal, <250 μg/dL)] were observed. Considering the course of the disease, the results of studies conducted to date, and the serological confirmation of a history of SARS-CoV-2 infection, PIMS-TS was suspected. The patient was transferred to the Department of Pediatrics for further treatment. According to current recommendations, intravenous infusions of human immunoglobulin [2 g/kg (i.e., 80 g of the preparation)] and ASA were administered. The course of the immunoglobulin infusion was uneventful. Due to the inserted drain and increased risk for bleeding, the recommended dose of ASA was reduced. Considering the boy's condition after two surgeries, intravenous antibiotic therapy with cefotaxime and metronidazole was continued, and amikacin was discontinued. While in the Department of Pediatrics, the patient experienced gradual improvement in his clinical condition. His vital signs, including blood pressure, were normal. The boy experienced fever during the first 2 days of hospitalization. An albumin infusion was performed in response to persistent hypoalbuminemia. Due to severe hypokalemia, simultaneous oral supplementation and drip infusion with potassium was performed, which achieved normokalemia on day 4 of hospitalization in the pediatric ward. In the days following the hospital stay, the boy's skin lesions gradually disappeared and his appetite improved significantly. In addition, the boy was receiving high-calorie “nutri-drink” preparations, which he tolerated well. Four days after laparotomy, the drain from the abdominal cavity was removed. Follow-up abdominal ultrasound revealed less fluid in the abdominal cavity and thinner walls of the large intestine. At the same time, gallbladder examination revealed thickened bile, with a slightly thickened wall and discreetly increased echogenicity in both kidneys. Lung ultrasound revealed the presence of fluid in the right pleural cavity up to 10 mm, in the left up to 5 mm, and fluid in the pericardial sac up to 5 mm at the widest point. Electrocardiography revealed sinus rhythm and non-specific ST segment abnormalities. Ursodeoxycholic acid was included in the treatment regimen due to characteristics suggestive of non-calculus cholecystitis. In subsequent laboratory investigations, a trend toward normalization of CRP level was observed. Peripheral blood counts revealed leukopenia (3.47 × 10^3^/μL), with slight neutropenia (1.28 × 10^3^/μL), and normalization of the lymphocyte count (1.54 × 10^3^/μL). An increase in hemoglobin concentration (10.9 g/dL) was observed. Creatine phosphokinase concentration was normal, and albumin concentration normalized. Troponin T and CK-MB concentrations were normal, as was urinalysis. Two days after the fever resolved, the dose of ASA was reduced to 300 mg daily (6.5 mg/kg body weight). Due to the necessity of performing echocardiography and further cardiological treatment on day 13 of hospitalization, the patient was transferred to the Department of Cardiology of the Medical University of Warsaw (Warsaw, Poland) for further treatment (Table 2).

**Table 2:** Patient's hospitalization timeline.

**Day of hospitalization**	**Patient condition and sympthoms**	**Laboratory tests**	**Imaging tests**	**Treatment**
1	Symptoms of appendicitis.	CRP 66.8 mg/LLeukocyte count 4.04 × 10^3^/μLNeutrophilic smear 70.7%Lymphopenia (0.77 × 10^3^/μL)	Presence of vesicular fluid and thickening of the cecum wall	Emergency laparotomy was performed: intraoperatively, an unchanged appendix and a significant amount of serous fluid were found.Cefazolin as antibiotic prophylaxis was administered
2	Watery stools, low-grade fever, persistent pain abdominal complaints	Serology for yersiniosis feces for culture		Antibiotic modification: cefotaxime was administered
4	Single ring-shaped skin lesion was observed			Second-generation antihistamine (levocetirizine)
5	A fever of >39°C	An increase in inflammatory marker levels (CRP, 137.6 mg/dL; procalcitonin, 1.96 ng/mL), with normal peripheral blood counts and leukocytes count, neutrophil smear of 86.2%, signs of normocytic anemia (hemoglobin, 10.4 g/dL; mean corpuscular volume, 79.5 fL), and normal platelet count	An increased amount of fluid with increased echogenicity in the bladder area, thickened walls of the cecum and terminal intestine, and enlarged mesenteric nodes (up to 20 × 14 mm) in the area of the removed appendix.	Metronidazole and intravenous amikacin was additionally administered
6			CT traces of fluid in the pleural cavities, the presence of fluid in the peritoneal cavity, interloop and bladder fluid (~150 mL), thickened walls of the cecum (up to 19 mm), thickened wall of the end intestine (up to 8 mm), without the presence of free gas in the peritoneal cavity, and numerous lymph nodes at the cecum and on the iliopsoas muscle (up to 28 mm)	Relaparotomy: a large amount of slightly cloudy serous fluid was aspirated from the peritoneal cavity, which was sent for culture (negative), and a drain was inserted into the bladder area. Intraoperative images of the intestines did not concur with the ultrasound and CT descriptions
7	Fever persisted, a reddened on the skin of the trunk. The annular lesions intensified and fused to form a “garland,” patient developed bilateral non-pyrogenic conjunctivitis, as well as chapped and reddened lips transferred to the Department of Pediatrics	Carcinoembryonic antigen and alpha-fetoprotein) levels = *N*, high positivity for SARS-CoV-2 antibodies [cut-off index (COI), 102 (normal, <0.99)CRP, 176.1 mg/L; procalcitonin, 2.97 ng/mL; ferritin, 301 ng/mL (normal, <124 ng/mL) D-dimer, 4,460 μg/dL (normal, <250 μg/dL)] hypokalemia, hypoalbuminia, INR 1.34 (normal, 0.85–1.25); D-dimer, 4,460 μg/dL hypokalemia		PIMS-TS was suspected human immunoglobulin [2 g/kg (i.e., 80 g of the preparation)] and ASA were administered intravenous antibiotic therapy with cefotaxime and metronidazole was continued, and amikacin was discontinued oral supplementation and drip infusion with potassium
11	Drain from the abdominal cavity was removed		Less fluid in the abdominal cavity and thinner walls of the large intestine. Lung—presence of fluid in the right pleural cavity up to 10 mm in the left up to 5 mm, and fluid in the pericardial sac up to 5 mm at the widest point	Ursodeoxycholic acid was included
13	No fever, no skin lesions, good appetite	Normalization of inflammatory markers		Due to the necessity of performing echocardiography and further cardiological treatment on day 13 of hospitalization, the patient was transferred to the Department of Cardiology of the Medical University of Warsaw (Warsaw, Poland) for further treatment.

## Discussion

Multisystem inflammatory syndrome (MIS-C) is a life-threatening condition occurring in children. It is most frequently post-infectious, rather than related to acute SARS-CoV-2 infection. Gastrointestinal symptoms are the most common clinical manifestations of MIS-C (87% of children), followed by muco-cutaneous (73%), cardiovascular (71%), respiratory (47%) and neurologic symptoms in 22% (9). In the first published paper reporting MIS-C 100% of presented patients had gastrointestinal symptoms (10). Similar results were presented in the material published by the group from Italy where 6 out of 10 patients presented gastrointestinal symptoms (11). In presented case report the first symptoms were worsening abdominal pain and low-grade fever, what is very similar to other cases. Also Periyakaruppan presents a case with a boy who manifested gastrointestinal symptoms and fever (12). However, laparotomy was not performed because CT abdomen revealed normal appendix. Similar to the authors' practice, the use of intravenous immunoglobulins as a treatment method quickly improved the patient's condition.

Our experience and the experiences of other authors should increase the awareness of surgeons who treat children with abdominal pain and suspected appendicitis during the Sars CoV 2 pandemic (13). In multisystem inflammatory syndrome the patients condition can rapidly deteriorate. Valitutti et al. based on their own experience, encourages the assessment of the activity of the heart muscle before the operation of exploring the abdominal cavity. A preliminary assessment of troponin, BNP, D-Dimer, ferritin and echocardiography can help to establish a precise differential diagnosis in children with acute abdomen, especially when in MIS-C evolution toward cardiogenic shock should not be neglected (14). Another recomendation is presented by Khesrani et al. They advice that in atypical abdominal pain syndrome during this pandemic an abdominal CT angio-scan should be performer to look for vascular damages in order to establish an appropriate medical treatment (immunoglobulins, corticoids) (15). In presented case none of above were performed before patients surgery. However, after analyzing the literature and acquiring our own experience, we apply the presented recommendations in our center.

## Conclusion

Due to the large number of illnesses caused by SARS-CoV-2 infection in recent months, the diagnosis of PIMS-TS/MISC should be considered in the differential diagnosis of acute abdominal symptoms, especially in atypical courses and interviews indicating exposure to SARS-CoV-2.

## Data Availability Statement

The raw data supporting the conclusions of this article will be made available by the authors, without undue reservation.

## Ethics Statement

Written informed consent was obtained from the minor(s)' legal guardian/next of kin for the publication of any potentially identifiable images or data included in this article.

## Author Contributions

BJ, MS-S, PH, and JS contributed to conception and design of the study. MS-S organized the database. BJ wrote the first draft of the manuscript. JS, EB, JK, EW, MT-M, and JC wrote sections of the manuscript. All authors contributed to manuscript revision, read, and approved the submitted version.

## Conflict of Interest

The authors declare that the research was conducted in the absence of any commercial or financial relationships that could be construed as a potential conflict of interest.
